# Strategies to Mitigate Greenhouse Gas (GHG) Emissions from the Solid Waste Management Sector: A Case Study of Vavuniya, Sri Lanka

**DOI:** 10.1155/2024/7709721

**Published:** 2024-09-16

**Authors:** Sobana Kayanan, B. F. A. Basnayake, R. T. K. Ariyawansha

**Affiliations:** ^1^ Department of Bio-Science Faculty of Applied Science University of Vavuniya, Vavuniya, Sri Lanka; ^2^ Postgraduate Institute of Science University of Peradeniya, Peradeniya, Sri Lanka; ^3^ Solid Waste Management Research Unit Department of Agricultural Engineering Faculty of Agriculture University of Peradeniya, Peradeniya, Sri Lanka; ^4^ Sustainable Environment Research Group Department of Environmental Technology Faculty of Technology Sri Lanka Technological Campus, Padukka, Sri Lanka

## Abstract

The waste sector is a substantial source of GHG emissions worldwide. Open dumping and internal combustion (IC) waste collection vehicles are significant sources of GHG emissions in Vavuniya. This research aims to estimate GHG emissions and recommend strategies to reduce emissions from the solid waste management sector. The IPCC methodology, considering Tier 1 estimation values based on default activity data, was used to estimate CH_4_ emissions from solid waste disposal sites. GHG emissions from collection vehicles were calculated based on IPCC mobile combustion recommendations. Three recommended strategies were considered based on demand, economic, and environmental feasibility and are expected to commence in 2025. According to current practices, open dumping generated 29.217 Gg of CO_2_ equivalent up to 2023, projected to rise by 37.8% by 2040. There will be a 57% decrease in open dumping-related GHG emissions by 2040 if composting is made mandatory for biodegradable waste, even though it produces emissions. Solar panels will be used to charge electric vehicles that will replace IC ones to cut emissions and fuel expenditures by 2025. The carbon sink reforestation program at the district level would need to begin in 2025 with an area of 161 hectares to sequester cumulative GHG emissions from composting dumpsites and fuel vehicles to achieve carbon neutrality by 2040. Investments from Vavuniya Carbon Sink Bonds (VCSB) on additional solar panels will ensure financial feasibility, having an internal rate of return of 23.18%. It paves the path to reducing GHG emissions, which is highly emphasized in the Nationally Determined Contributions, National Climate Change Policy, and waste management policy of Sri Lanka.

## 1. Introduction

Increasing population, thriving economy, urbanization, and growing living standards increase Municipal Solid Waste (MSW) generation in developing countries [1]. The inadequate management of MSW has emerged as a significant concern for the governments of several Asian and African nations [2]. In developed countries, waste management is rigorously adhered to by established rules, laws, and policies. In developing countries, over 50% of waste is improperly discarded, contributing to GHG emissions and environmental damage [3]. GHG is the primary driver of global warming and climate change, which disrupts the ecological balance [4]. The production of GHG from anthropogenic activities is a crucial worldwide concern regarding environmental health [5]. The MSW sector accounts for roughly 5% of the worldwide GHG budget [6]. CH_4_, N_2_O, and CO_2_ are the key reported GHG emissions from the waste sector [7, 8].

It is essential to reduce the GHG emissions in the Solid Waste Management (SWM) system to achieve the statements addressed in the Nationally Determined Contributions (NDC) of Sri Lanka, which was submitted to the United Nations Framework Convention on Climate Change (UNFCCC) and National Climate Change Policy of Sri Lanka for the emission reduction [9, 10]. Over the last two decades, dumpsite rehabilitation efforts have improved waste segregation, collection, composting, and recycling [11, 12]. However, in the Vavuniya district, the conventional practices have been continued where there is no notable enhancement in SWM. Open dumping is carried out by the Local Authorities (LAs) of Vavuniya, which is considered a primary anthropogenic methane emission source and operates in four locations within the district [13]. Fuel combustion from solid waste collection vehicles produces GHG emissions, causing global warming and air pollution [14]. In this regard, the LAs have to make considerable efforts to reduce emissions at each LA level. This study aims to reduce GHG emissions in the SWM sector in Vavuniya by introducing composting as an alternative to the open dumping of biodegradable waste. Vehicle electrification was proposed; vehicles must be charged with solar panels instead of fuel to reduce emissions. A carbon sink reforestation program is suggested to offset cumulative GHG emissions from the selective SWM activities. Moreover, technical and financial feasibility was undertaken to promote Vavuniya Carbon Sink Bonds (VCSB) to implement the project.

## 2. Methodology

### 2.1. Study Area

**Figure 1:**
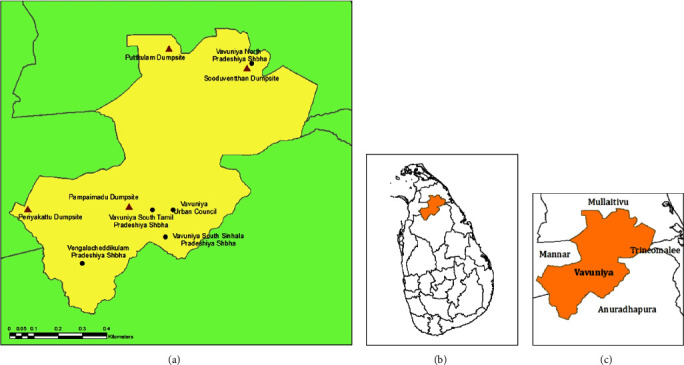
The (a) figure shows the LAs and dumpsites in the Vavuniya district, the (b) figure shows the location of the Vavuniya district, and the (c) figure shows the surrounding districts of Vavuniya [15].

**Figure 2:**
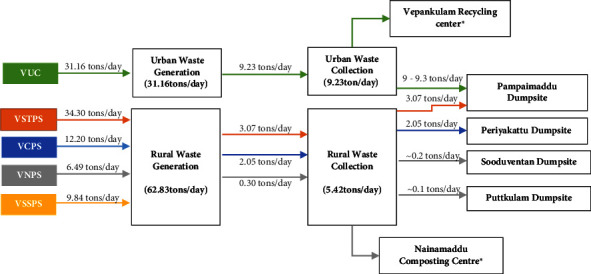
Solid waste flow in Vavuniya district. ^∗^Both recycling centers received very limited amount of waste ∼0.3 tons per day occasionally due to the limited space availability and labour force.

The current study was conducted in the Vavuniya district, which is located in the Northern Province of Sri Lanka, and is a connecting hub of the North Central and Eastern Provinces of Sri Lanka. Five LAs that are functioning in the Vavuniya district, namely, Vavuniya Urban Council (VUC), Vavuniya South Tamil Pradeshiya Shaba (VSTPS), Vengalacheddikulam Pradeshiya Shaba (VCPS), Vavuniya North Pradeshiya Shaba (VNPS), and Vavuniya South Sinhala Pradeshiya Shaba (VSSPS) (Figure 1), whereas the LAs are further categorized into ward places presented in Table 1. The highest population density in the VUC is nearly 2017 per sq. Km, whereas the lowest is VNPS [16, 17]. Four LAs provide solid waste management services in Vavuniya except for VSSPS. Mixed types of waste collection, limited recycling and recovery activities, and the open dumping of waste (Figure 2) have been considered as the major improper practices at the district level where anticipated environmental issues.

### 2.2. Data Collection

The study was based on a project to formulate solid waste management plans. Strategies to curtail GHGs, promote NDCs, and sustainable development goals are part of the plans. Preliminary data on SWM were gathered via a desk examination of secondary sources. Pertinent data and information on GHGs were extracted from a questionnaire survey that was designed under eight main topics: institutional structure, infrastructure development, research and development and education and training, social development, environmental management, resource allocation, monitoring and risk assessment, disaster management, and regulatory and law enforcement to collect comprehensive solid waste information [15]. The questionnaires were given to the LAs prior to the discussions on the information provided by the responsible officers and workers of the respective LAs. The district engineer, five secretaries of LAs, five technical officers, five public health inspectors, an environmental officer, seven supervisors, and ten workers provided data and information on solid waste compositions and quantities, waste collection vehicles, fuel consumptions, and disposal facilities. Moreover, the collection routes, frequencies, vehicle breakdowns, repair and maintenance, and information on collection and disposal crews were obtained. Site visits were made to quantify some of the data, like waste recycling, disposal amounts, and waste degradation in dumpsites. The data and information obtained from the Vavuniya district were compared with those of other districts and provinces to determine the outliers by researching such variations.

### 2.3. Estimation of GHG Emissions

Two approaches were considered: Business As Usual (BAU) and Recommended Strategies (RS) for GHG emission reduction. The open dumping of degradable waste and solid waste vehicles are considered the key GHG emission sources at the LAs of Vavuniya. The BAU was developed to estimate the GHG emissions as a continuation of current practices where emissions from open dumping and solid waste vehicles were considered. Instead of BAU, the economically and environmentally feasible Recommended Strategies “RS” were considered for the GHG emission reduction in the SWM sector in Vavuniya and is expected to commence in 2025, as presented in Table 2.

In the IPCC methodology, estimates of emissions and removals are categorized by sector. These sectors encompass homogeneous activities, sources, and sinks. The sectors include Energy, which is divided into Fuel Combustion and Fugitive Emissions from Fuels; Industrial Processes and Product Use (IPPU); Agriculture, Forestry, and Other Land Use (AFOLU); Waste; and another category, which includes indirect emissions from nitrogen deposition from nonagriculture sources. The IPCC methodology employs three tiers for emissions' estimation: Tier 1, Tier 2, and Tier 3. Tier 1 is the simplest approach, Tier 2 is intermediate, and Tier 3 is the most complex and data-intensive, typically providing the highest accuracy. Due to limited data availability, the Tier 1 method was adopted. This approach leverages readily available national or international statistics, default emission factors, and supplementary parameters, ensuring its feasibility for all countries [19].

In the solid waste management sector, the haulage vehicles belong to the IPPU sector. Although IPCC guidelines are geared towards determining key source categories of existing systems, they are essential and can address novel and transitional technologies for solar-driven systems. Hence, other criteria must be considered when determining key source categories that are not as easily accessed through a quantitative analysis. These criteria include the following.

#### 2.3.1. Mitigation Techniques and Technologies

In the case of the study, the mitigation technologies are composting and replacing IC haulage vehicles with electric ones. There is certainty in identifying key source categories; thus, the Tier 1 methodology can be used because there are adequate data on waste dumping and the reductions of CH_4_ emissions in the future with composting. Similarly, fuel consumption is known; thus, emissions from the past can be quantified, and none will be in the future because of electric vehicles.

#### 2.3.2. High Expected Emission Growth

At the end of the IC and electric vehicle lifecycle, there are uncertainties in determining the emissions, and they could be key sources of emissions. This study assumes that both negate each other in terms of emissions because IC engines and most parts of the vehicles are discarded at the end of life in Sri Lanka.

#### 2.3.3. High Uncertainty

There is high uncertainty in using lithium-ion batteries because nonrecyclability becomes a key source of emissions since mining to extract lithium ore requires fossil fuels. Unless mining is done with electric vehicles and if there is a demand for secondary use of the batteries, such as for nonmovable energy storage, therefore, a Tier 2 application is needed to quantify much detailing of the lifecycle from extraction to recycling.

#### 2.3.4. Unexpectedly Low or High Emissions

Alternatively, less efficient lead-ion batteries are a low source of emissions. However, with increased demand at a lower energy density than lithium, it may become the key source of emissions. In the present-day context, lead-ion batteries can be easily produced and recycled using solar power and belong to Tier 1 estimation.

### 2.4. The “BAU” Strategy

#### 2.4.1. Solid Waste Disposal Sites

The dumpsites are a significant source of GHG emissions, especially CH_4_ [20, 21]. In Sri Lanka, 85% of the disposal sites are dumpsites [22]. In Vavuniya, four dumpsites are used for solid waste disposal, considered the key source of GHG emission [15] in the waste sector. The IPCC Methodology, considering Tier 1 estimation values based on default activity data, was used to estimate the CH_4_ emission from Solid Waste Disposal Sites (SWDS) using equations (1)–(6). The origin of biogenic CO_2_ was not addressed in the waste sector. Other emitted gases from dumpsites, such as nonmethane volatile organic compounds (NMVOCs), nitrous oxide (N_2_O), nitrogen oxides (NO_x_), and carbon monoxide (CO), can be negligible [23].

*(1) Estimation of Decomposable Degradable Organic Carbon at the Inventory Year T*. Equation (1) defines Decomposable Degradable Organic Carbon (DDOCmd) (T) at the inventory year T. The deposited waste at year T is W (T) in Gg; Degradable Organic Carbon (DOC) as a fraction of Gg of C/Gg of waste is 0.15 for bulk municipal solid waste as IPCC default value. DOC_f_ is the fraction of DOC decomposing under anaerobic conditions (default value: 0.5). The Methane Correction Factor (MCF) for uncategorized SWDS is 0.6, as defined by IPCC.

(1) $$\begin{array}{c} \text{DDOCmd}\left(T\right)=W\left(T\right)\times \text{DOC}\times {\text{DOC}}_{f}\times \text{MCF}. \end{array}$$

*(2) The Total Amount of DDOCm Decomposed in the Year T*.

(2) $$\begin{array}{c} \text{DDOCmrem}\left(T\right)=\text{DDOCmd}\left(T\right)\times e^{\left(-k\times \left(13-M\right)/12\right)}. \end{array}$$

Equation (2) determines the remaining decomposable degradable organic carbon from solid waste at the end of year *T*, where the values for rate of the reaction content (*k*) and month of the reaction start (*M*) are 0.09 and 06 based on the IPCC waste model. The decomposed DDOCmd (*T*) and accumulated DDOCmd (*T*) in the year of *T* are expressed by equations (3) and (4), where the total amount of DDOCm decomposed in year *T* is estimated using equation (5).

(3) $$\begin{array}{c} \text{DDOCmdec}\left(T\right)=\text{DDOCmd}\left(T\right)\times \left(1-e^{\left(-k\times \left(\left(13-M\right)/12\right)\right)}\right), \end{array}$$

(4) $$\begin{array}{c} \text{DDOCma}\left(T\right)=\text{DDOCmrem}\left(T\right)+\left(\text{DDOCma}\left(T-1\right)\times e^{-k}\right), \end{array}$$

(5) $$\begin{array}{c} \text{DDOCmdecomp}\left(T\right)=\text{DDOCmdec}\left(T\right)+\left(\text{DDOCma}\left(T-1\right)\times \left(1-e^{-k}\right)\right). \end{array}$$

*(3) Methane Generation at the Inventory Year T*.

(6) $$\begin{array}{c} {\text{CH}}_{4}\text{ generated}\left(T\right)=\text{DDOCmdecomp}\left(T\right)\times F\times \frac{16}{12}. \end{array}$$

Finally, methane generation at the inventory year (*T*) was estimated in equation (6) from the decomposed waste, which is denoted as DDOCmdecomp (*T*), where the fraction of CH_4_ (F) is 0.5. The molecular weight ratio of methane/carbon is 16/12.

*(4) Estimation of GHG Emissions in 2040*. To estimate the GHG emission in 2040 at the dumpsites, equation (7) was used to calculate the estimated population in 2040 [24], where the solid waste generation and solid waste collection were estimated using equations (8)–(10).

(7) $$\begin{array}{c} P_{\left(2040\right)}=P_{0}\times e^{\text{rt}}, \end{array}$$

(8) $$\begin{array}{c} {\text{SWG}}_{2040}=P_{2040}\times {\text{WG}}_{P}, \end{array}$$

(9) $$\begin{array}{c} {\text{SWC}}_{2040}={\text{SWG}}_{2040}\times {\text{WC}}_{\text{ef}}, \end{array}$$

(10) $$\begin{array}{c} {\text{WC}}_{\text{ef}}=\frac{{\text{SWC}}_{2023}}{{\text{SWG}}_{2023}}. \end{array}$$

Equation (7) estimates the population in 2040, representing the estimated population as P_2040_, *P*_0_ as the initial population [7], *e* as the Euler number (2.7182), population growth rate *r* is 1.3, and *t* as the time in years [25]. Equation (16) calculates Solid Waste Generation (SWG) in tons per day, with WG_p_ denoting per capita per day waste generation where UC and PS have 0.6 kg and 0.4 kg, respectively [26]. Solid waste collection (SWC) is estimated through equation (9), where WC_ef_ represents Waste Collection Efficacy given in equation (10). The proportion of solid waste compositions was assumed to be the same in 2023 and 2040 when the GHG emissions were determined using equations (1)–(6).

#### 2.4.2. Solid Waste Transportation Vehicles

Transportation of waste from its source to its destination for treatment or disposal contributes to GHG emissions. CO_2_, CH_4_, and N_2_O are emitted into the environment when waste transportation vehicles powered by fossil fuels such as diesel and gasoline are utilized [27, 28]. Diesel-powered vehicles have been used for collection and transportation in Vavuniya [18]. According to IPCC recommendations [29], equation (7) was used to arrive at the expected total GHG emissions from the combustion of fossil fuels. Equation (8) is used for the energy required by vehicles during solid waste collection and transportation.

(11) $$\begin{array}{c} E_{T}={\text{FC}}_{Y}\times D_{\text{FT}}\left({\text{EF}}_{{\text{CO}}_{2}}+\left(25\times {EF}_{{\text{CH}}_{4}}\right)+\left(218\times {\text{EF}}_{N_{2}O}\right)\right), \end{array}$$

(12) $$\begin{array}{c} {\text{EC}}_{V}={\text{FC}}_{d}\times D_{\text{FT}}\times \left(\frac{1}{3.6}\right)\times 6. \end{array}$$

GHG emissions from diesel combustion (*E*_*T*_) were computed in tons of CO_2_ equivalent per year using equation (11), where FC_Y_ is the fuel consumption in liters per year. The diesel fuel thermal (D_FT_) is 38.136^∗^ 10^−6^ TJ/L [30]. The IPCC's default figure for EF_CO2_ is 74,100 kg/TJ, while EF_CH_4__ and EF_N_2_O_ are 3.9 kg/TJ. The CH_4_ and N_2_O are comparable to 25 and 218 CO_2_ [31]. The Energy Consumption of the Vehicles (ECv) was determined in kW per day using equation (12). Diesel Fuel Thermal (D_FT_) has a reference value of 38.136 MJ/L [30]. The FC_d_ is Fuel Consumption per day in liters, converted into kWh and kW per day, assuming six running hours per day.

GHG emissions from solid waste collection vehicles from 2000 to 2023 were calculated based on equation (11) at each LA level, while the expected GHG emission in 2040 was calculated using equations (19) and (20). FR represents fuel requirement in liters per ton, while FC stands for fuel consumption measured in liters per day.

The energy consumption was calculated using equation (12) based on the FC in 2040 and FC in 2023.

(13) $$\begin{array}{c} \text{FR}=\frac{{\text{FC}}_{2023}}{{\text{SWC}}_{2023}}, \end{array}$$

(14) $$\begin{array}{c} {\text{FC}}_{2040}=\text{FR}\times {\text{SWC}}_{2040}. \end{array}$$

### 2.5. GHG Emission Reduction Strategies

#### 2.5.1. Solid Waste Composting

Solid waste composting is considered a viable alternative to the open dumping of degradable waste, which is expected to commence in 2025. Vavuniya is an agriculture-based area with a demand for compost [17]. Solid waste composting is the best option for GHG emission reduction when compared to open dumping [32]. The IPCC guideline includes CH_4_ and N_2_O emissions estimation from composting based on equations (15) and (16). Biogenic CO_2_ emissions are excluded [33].

(15) $$\begin{array}{c} {\text{CH}}_{4}\text{ Emission}=M_{\text{ow}}\times {\text{EF}}_{\text{CH}4}\times 10^{-3}, \end{array}$$

(16) $$\begin{array}{c} N_{2}O\text{ Emission}=M_{\text{ow}}\times {\text{EF}}_{N2O}\times 10^{-3}. \end{array}$$

The *M*_ow_ is the amount of organic waste (in Gg) processed in composting; EF is the emission factor in *g* of CH_4_/kg and *g* of N_2_O/kg of waste treated as 4 and 0.24, respectively, based on the IPCC default values. Total emission was estimated and compared in Gg of CO_2_ equivalent where methane and N_2_O are 25 and 298 times higher than CO_2_ [34].

#### 2.5.2. Carbon Sink Reforestation Program (CSRP)

Trees have the capability of carbon sequestration, which aids in lowering the CO_2_ levels in the atmosphere [35]. Equation (17) was applied to determine the CO_2_ sequestration potential (CS_*T*_) in kilograms per hectare at the year *T*.

(17) $$\begin{array}{c} {\text{CS}}_{T}={\text{CG}}_{T}\times \left(\frac{44}{12}\right)\times {\text{SF}}_{T}\times \text{NT}. \end{array}$$

The Number of Trees (NT) per hectare or the stocking density was considered to be 3100 trees per hectare [36]. Moderate cumulative growth (CG_*T*_) in kg carbon/tree at the year *T* multiplied by 44/12 to be converted as kg CO_2_ sequestrated at the year *T* where the SF_*T*_ is the moderate survival factor at the year *T*. The default values CG_T_ and SF_T_ are obtained from the method for calculating carbon sequestration by the US Department of Energy [37]. Equation (18) revealed that the Required Area (RA_H_) in hectares was computed to neutralize the emission by 2040 at each LA and district level.

(18) $$\begin{array}{c} {\text{RA}}_{H}=\frac{\left({\text{GHGE}}_{\text{SWDS}}+{\text{GHGE}}_{C}+{\text{GHGE}}_{V}\right)}{{\text{CS}}_{2040}}. \end{array}$$

The GHG emissions from solid waste disposal sites (GHGE_SWDS_), composting (GHGE_C_), and solid waste collection vehicles (GHGE_V_) up to 2040, which is divided by the CS up to 2040 to get the required area in hectares at each LA level.

#### 2.5.3. Electrification of Vehicles

The gasoline vehicles are intended to be substituted by electric vehicles by 2025 to reduce GHG emissions from solid waste collection vehicles. It can be expressed as

(19) $$\begin{array}{c} \text{NV}={\text{EC}}_{v}\times \frac{\left(1-E_{f}\right)}{\text{VC}}\times O_{p}, \end{array}$$

(20) $$\begin{array}{c} R_{\text{SP}}=\frac{\text{ECv}}{C_{\text{sp}}}\times P_{\text{SH}}. \end{array}$$

NV is the number of vehicles needed for solid waste collection per day or the number of trips per day expressed in equation (19). EC_v_ is the energy consumption of fuel vehicles (ECv) in kW per day, where Ef is the efficiency of the electric vehicles, which is 0.6 [38]. VC is the vehicle capacity in kWh, and the *O*_*p*_ is the operation hours of the vehicle per day. Equation (20) was used to compute the required solar panels (R_SP_) to charge electric vehicles, which were calculated using ECv in kW per day, the capacity of the solar panel in kWh, and the peak sunshine hours P_SH_ is considered six hours per day.

### 2.6. Technical and Financial Evaluation

#### 2.6.1. Technical Evaluation

The carbon footprint in the manufacturing of batteries is a concern. Therefore, it is crucial to select the best and most suitable one for recycling by considering the end-of-life option. Moreover, the selection of a suitable prime mover is important for the project.

#### 2.6.2. Financial Evaluation

Most of the investments for activities in the LAs in the past relied on direct government initiatives through budgetary allocations and project-based activities by Non-Governmental Organizations (NGOs), International Agencies (INGOs), and foreign loans. LAs paid for some of the foreign-funded loans depending on the agreements. Also, LAs take loans from local government banks to purchase solid waste collection vehicles to be repaid with income from local tax and government allocations. In the present context, it is very difficult and challenging to get foreign-funded loans or grants and there are many restrictions on obtaining foreign exchange to purchase machinery and equipment. Nevertheless, there are many bilateral agreements to implement NDCs. It is understood that projects must be technically and financially feasible for both parties to agree on the required investment. It is also expected that the local party should invest to increase the viability of the project in terms of responsibility and sustainability. Therefore, each of the project components should contribute towards fulfilling the NDCs. There should be sensible financial assumptions to make the project viable. They are

- The electrification of vehicles will increase and improve waste collection coverage.
- The project will not fail with 30% less income from solar power generation.
- The proposed system will feed the national grid with a net accounting agreement option.
- The Ceylon Electricity Board will not reduce the agreed tariff, unless to reach very low inflation in the country.
- The value of the existing stock of IC engine vehicles is not accounted in the financial evaluation.
- The establishment of the plant propagation laboratory and nurseries will generate income as Vavuniya Carbon Sink Bonds (VCSB). VCSB will be invested in solar power generation to pay back the loan and interest. People living or working in the Vavuniya district will be given priority in purchasing VCSB. Most importantly, home garden bondholders are the key stakeholders. They will benefit from the solar electrification project because the LAs will reinvest the profits in their household solar rooftops. Therefore, the adult population, assumed to be just over 90,000 being 50% of the population, will participate in the bond scheme. It will include large numbers from the urban sector. The bonds can also be purchased to improve the waste collections and offset the amount from taxes.
- Operational and financial statement of assumptions

It is imperative to dwell on the statement of limiting conditions. They can be listed as

(1) The government approvals will be given to implement the project partnering with or without foreign collaboration.
(2) The assets can be insured against all forms of disasters.
(3) The project will be able to adhere to the project implementation timeline.
(4) The technical issues before commencing of the project can be solved. A small pilot study will be undertaken with the selected enterprises to evaluate the performances of the solar system. An independent body and interested financial institutions will be requested to participate in the evaluation. It can be a technical and financial evaluation team with stakeholders from the district, LA officials, and officials of the Central Government.
(5) The expected expenditure and revenue are the basis for the financial projections reported. There are three capital cost components. They are (A) purchase of electric prime movers, (B) solar panels, and (C) establishment of plant propagation laboratory and nurseries. Apart from the capital costs, VCSBs are promoted to offset the capital costs of (C) by way of additional solar panels to generate income for CS activities. The later VCSB value is derived based on the ADB funded expenditure of USD 25 million for developing and improving 53,075 ha [39]. The present-day value is worked out based on a base inflation rate of 4.5% in fossil fuel [40] increase compounded over 20 years. The cost of solar panels is based on the present market price of USD 11,589 for 20 kW so that they can be installed in rooftops rather than have solar farms. According to the Federal Office of Energy Efficiency and Renewable Energy, in the USA, the estimated scheduled maintenance costs for an electric vehicle average $0.06 cents per mile, while it is at $0.10 per mile for a conventional ICE-powered ride [41]. Additionally, waste management vehicles need more cleaning. Therefore, a value of 25% of the fuel allocation is used as expenditure. Moreover, 3% of the revenue will be used to maintain PV panels and as administration costs. The value of the rupee is expected to reduce by 5%, thus inflation of 5%. Straight line depreciation over 20 years is used to have an economic life of 10% of the purchase price for equipment and 50% for solar panels. The general insurance is 0.065% of the premium for machinery and equipment. There are many options for solar power generation agreements. This study considers a fixed revenue of USD 0.12 per kWh unit for the net accounting system. In a recent government circular, the tariff has been increased from USD 0.12 to 0.16 for promoting investments [42]. It has been decided to have four streams of investments, notably,
  - The excess generations from the solar panels
  - The allocation of fuel is used as an investment. In the first year of operation, there will be generations for nine months of the project year. In the following years, for four years, solar installations from fuel allocations will continue for this financial evaluation.
  - The VCSB will be implemented, and the revenue generation will be effective after three months because of the envisaged installation timeline. Hence, there was power generation of nine months in the first year.
  - The investment in the carbon sink will be in the form of seed and vegetative propagation, including tissue culturing to grow healthy trees and plants for short- and long-term revenue generations. The VCSB stakeholders will participate in improving and developing home garden forests and community forests in the identified lands in the Vavuniya district. They can outright pay for the bonds, or it could be through a financial institution that will hold the bonds until the sale of the short-term produce like green gram. The project will provide the inputs for short-term income generation.

The assessment of the feasibility was evaluated using the financial indicators: revenue expenditure, retained profit, operating cash flow, project cash flow, tax is not considered in this financial because of investing the profits in solar rooftops, return of investments (ROI), internal rate of return (IRR), and payback period.

## 3. Results and Discussion

The solid waste generation, composition, and disposal of waste, the degree of economy, and the operation and management of each municipality can all be taken into consideration when selecting emission reduction strategies, such as raising the recycling rate, installing waste-to-energy conversion, benchmarking MSW, and establishing material recovery facilities for waste management. These strategies are crucial for growth planning and establishing goals for each municipality to lower emissions from the waste sector [38, 43–45]. The recommended emission reduction strategies were finalized based on demand, economic viability, and environmental sustainability presented in Table 2.

### 3.1. GHG Emission for “BAU” Strategy

#### 3.1.1. GHG Emissions from Disposal Sites

According to the current SWM practices, the GHG emissions were estimated up to 2040 from open dumpsites and the fuel combustion of vehicles. Four open dumpsites (Figure 1) are operated in the Vavuniya district to dispose of solid waste. The anaerobic decomposition of degradable waste at the dumpsites produces gases, mainly CO_2_, CH_4_, and N_2_O, contributing to global warming [46].

VUC and VSTPS have disposed of mixed solid waste in the Pampaimadu dumpsite since 2000. The VNPS has been operating dumpsites at two locations, such as Puttkulam and Sooduventhan, since 2016 to reduce transportation costs. In VNPS, there are two small towns located far away from each other where the population density is lower, which helped establish the disposal site in two locations. The VCPS has been operating the Periyakattu dumpsite since 2009. According to the 2023 records, the solid waste composition has a huge degradable portion at each LA level, with VUC having 72%, VNPS 75%, VCPS 60%, and VSTPS 67%. It was the major source of GHG emissions from each dumpsite.

**Figure 3:**
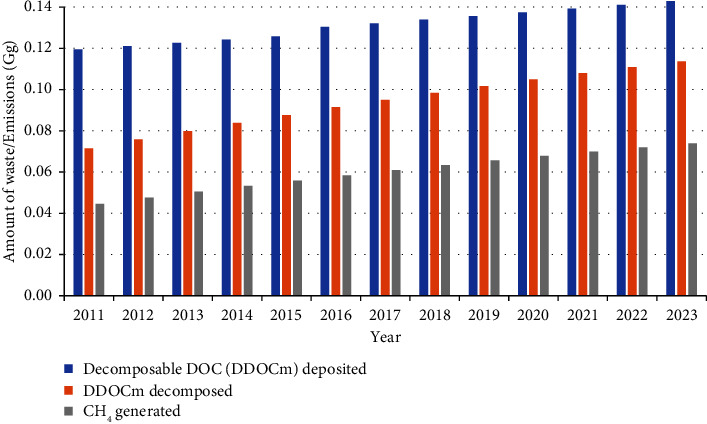
CH_4_ emission from SWDS.

The GHG emissions from SWDS were computed using the IPCC methodology of 2006 and 2019 refinement.  Figure 3 illustrates DDOCm deposition, decomposition, and CH_4_ emission from all dumpsites from 2011 to 2023. The GHG emission from each dumpsite until 2023 was computed using the equations (1)–(6) and presented in Table 3, along with the IPCC waste model and default values from IPCC guidelines for Tier (1) Emission Factor (EF). Table 3 demonstrates that the most significant contributor to GHG emissions is the Pampaimadu dumpsite.

To estimate the GHG emission in 2040 at the dumpsites, equation (7) was used to calculate the estimated population in 2040 [24], where the solid waste generation and solid waste collection were estimated using equations (8)–(10) at each LA level addressed in Table 4. The proportion of solid waste compositions was assumed to be the same in 2023 and 2040 when the GHG emissions were determined using equations (1)–(6) expressed in Table 3.

#### 3.1.2. GHG Emission from Solid Waste Collection Vehicles

GHG emissions from solid waste collection vehicles from 2000 to 2023 were calculated based on equation (11) at each LA level, while the expected GHG emission in 2040 was calculated using equations (13) and (14), where abbreviations and the values are defined in Table 5. The energy consumption was calculated using equation (12) based on the FC in 2040 and FC in 2023, displayed in Table 5.

### 3.2. Recommended Strategies for GHG Emission Reduction

#### 3.2.1. Solid Waste Composting

**Figure 4:**
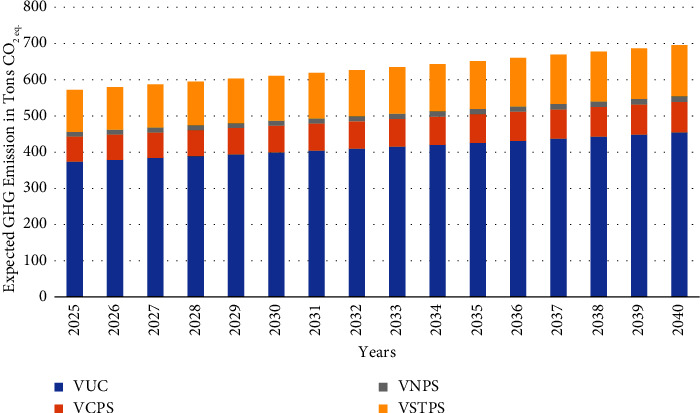
Expected GHG emission from composting.

Solid waste composting is expected to be fully established by 2025 at each LA level instead of open dumping of degradable waste. The solid waste collection was calculated using equations (7) and (10). The solid waste composition was considered per the 2023 information. The emissions from solid waste composting at each LA level were estimated using equations (15) and (16), shown in Figure 4.

**Figure 5:**
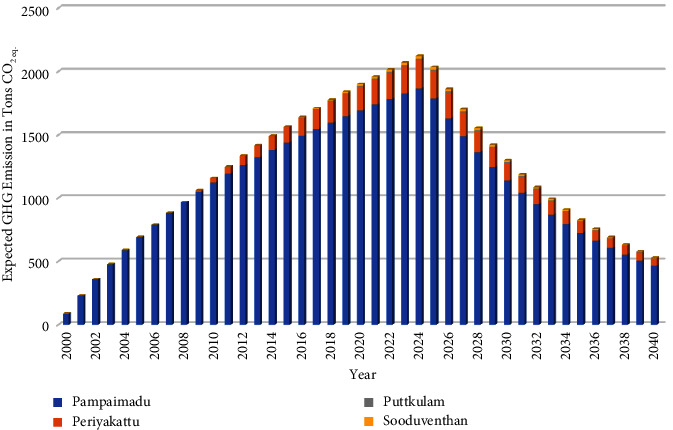
GHG emission from dumpsites.

First, SWDS emissions were projected to be reduced by implementing composting in 2025. Consequently, the predicted GHG emissions and reductions at each dumpsite in 2040 are outlined in Table 6, whereas Figure 5 depicts the GHG emissions from the dumpsites.

#### 3.2.2. Electrification of Vehicles

It was envisaged that diesel-powered vehicles would be replaced with 60 kWh electric tractors or compactors to reduce GHG emissions from solid waste collections. The number of required tractors was calculated based on equation (19) with the intention of charging the vehicles using 100 kWh solar panels, with the predicted electricity generations at each LA level determined using equation (20) presented in Table 7.

#### 3.2.3. Carbon Sink Reforestation Program (CSRP)

The Recommended Strategy (RS) anticipated to be implemented by 2025 is the introduction of composting and electric automobiles instead of open dumping of biodegradable and gasoline vehicles. The acceptance of existing methods referred to as “Business as Usual” (BAU) is contrasted with the adoption of RS in Table 8. The findings indicate that RS, which is anticipated to lower 48.13 percent of total GHG emissions by 2040, would reduce dumpsite emissions by 31.13 percent. The remaining cumulative emission is anticipated to be neutralized by the fully grown trees in the reforested lands beyond 2040. The needed area is computed using equations (11) and (12), shown in Table 8, for each LA level and district.

### 3.3. Technical Evaluation

There is considerable debate on the use of electric vehicles because of the high carbon footprint in manufacturing batteries [47]. Lead-acid batteries can be designed to be high power and are inexpensive, safe, recyclable, and reliable. However, low specific energy, poor cold-temperature performance, and short calendar and lifecycle impede their use [48]. Nevertheless, it is a viable option for small, slow-moving waste collecting tractors. Moreover, according to [49] “while nearly all -99% - of lead batteries are recycled, few lithium-ion batteries are recyclable, and the rate could be less than 5%. Most parts in electric vehicles are reusable, whereas the batteries are not designed to be recycled or reused.” “Once in landfills, metals from the batteries can contaminate water and soil.” The global carbon footprint of the lithium-ion battery industry is projected to reach up to 1.0 Gt CO_2_-eq per year within the next decade. With material supply chain decarbonization and energy savings in battery manufacturing, a lower estimate of 0.5 Gt CO_2_-eq per year is possible. Moreover, there are improved techniques for recycling lithium-ion batteries [50–54], thus making the more advanced option a must for developing nations. The second-hand market for batteries is relatively high, making the electric vehicles option very attractive. Interestingly, 33 kW range electric tractors capable of lifting 4000 kg are sold at competitive prices to diesel ones in India. In Sri Lanka, e-waste is categorized as hazardous waste. According to the National Environmental Act of 2008, every generator, collector, storer, transporter, recoverer, recycler, and disposer must obtain a license from the Central Environmental Authority (CEA) under Gazette Extraordinary 1534/18 [50, 51]. In Sri Lanka, licensed collectors have partnered with world-class battery recyclers in Belgium and Korea [53].

### 3.4. Financial Evaluation

The capital costs as shown in Table 9 amount to just over USD 600,000 with over 50% for solar generation panels. The electric tractors are reasonably priced, and the replacement of IC will allow the best performance with greater comfort for the operator. The need for compactors should be evaluated. Although the cost of establishing the nurseries is high, it is the backbone of the project. The compost of urban biodegradable wastes with the required additions of inputs such as *Arbuscular mycorrhizal* fungi (AMF) [55] and biochar [56] will have a ready market in the plant nurseries. The survival rate will be higher because of healthy plants grown in the nurseries for distribution. Additional investment is required in terms of VCSBs, amounting to USD 183,208. The bonds will be used to purchase solar rooftop PVs that will generate power to make the project viable. The VCSB is valued at USD 183,208 for 160 ha, which is USD 1145/ha, which exceeds the 2003 ADB project [39], having been compounded to give a value of USD 1135/ha. Each bond is valued at USD 2.00 derived from the maximum participation of the population in purchasing VCSB.

**Figure 6:**
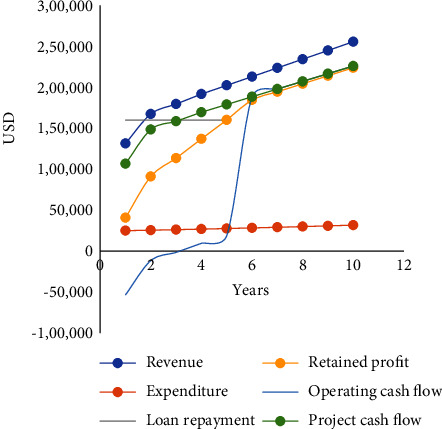
Financial variables indicate the changes with time.

The loan repayment is given in Table 10 in the appendix at an interest rate of 10% and the duration of the loan is five years. The annual payment is USD 160,052. The expenditure increased from USD 25,018 to USD 31,799 at the end of 10 years, as shown in Table 11 in the appendix. The revenue increases from USD 131,531 to USD 255,756 in 2034 as shown in Table 12 in the appendix. The working capital should be adequate for the sale of plants from USD 6,623 with more than 50% increase in 10 years as shown in Table 13 in the appendix. The profit and loss account given in Table 14 in the appendix indicate a gross profit of USD 106,512 in the first year (2025) and at the end of the 10^th^ year USD 223,957. The operating profit will increase by almost 60% at the end of 2030. The retained profit will likely be USD 160,267 in five years with a ROI of 21.39%. The payback period is four years, as given in the forecast of project cash flow, Table 15 in the appendix. There is an increase of 48% in the Operating Cash Profit at the end of ten years. Nevertheless, the Operating Cash Flow is inadequate in the first three years of operation. Hence, a grace period is needed for the loan repayment in the first year, thus making it possible for the second year's loan instalment and interest payments. It can easily be accommodated since Project Cash Flow increases by 60% in the fifth year. The project is viable with an IRR of 23.18%. Some financial variable indicators are shown in Figure 6. There are many ways to overcome negative operating cash flow. One way is to extend the loan repayment period to six or seven years or have a grace period of one year for both interest and loan repayment. That saving can be invested on more solar panels to generate more revenue after installation.

In the event of 30% power generation reductions, the IRR reduces to 13.44%. It is above the borrowing rate; hence, the project is still viable and recovering in the seventh year. It will sustain an interest of 9.52% in the first year's operating cash profit of USD 75,181. Such a scenario could be given to market long-term bonds, thus avoiding loans altogether or equity with VCSB. The project can be described as an investment catalyst for the development of the country. Notably, the administrators have adequate confidence to launch sustainable development since it is very advantageous. Because the feasibility of electrification is based on the concept of the LAs to save on diesel while investing in solar power generations, it will provide additional funds to purchase tractors and meet other expenditure. Solar income will be much above their need to invest in the Carbon Sink Reforestation Program. Certainly, it is a win-win situation for the waste generators, including bondholders, LAs, and the Central Government. LAs will not have to face the fuel increases every year crippling the budget, thus causing inevitable and uncontrollable mismanagement. Importantly, the project will never fail because the base inflation rate tends to zero with the reduction in the use of fossil fuels [40].

Moreover, plant nurseries will continue to increase the supply of plants to meet the demand because of the increase in the value of tropical forests for medicinal and home garden (agriculture) productions [57]. It is an attractive component in the investment cycle. Notably, Foreign Direct Investment (FDI) has an active role in the economic growth of the country [58]. Unfortunately, a decline persists in the availability of FDI for developing countries [59]. Moreover, the country is unable to repay debt-burdened loans. To make matters worse, the government is taking loans to buy fossil fuel to run the country. Notably, local banks can provide loans in Sri Lankan rupee but require foreign exchange to purchase the solar PVs and haulage vehicles. Bilateral trade agreements may be used for this purpose. Another more lucrative alternative is to tap the tourist sector to purchase VCSBs. Vavuniya carbon sink reforestation program will physically involve foreign bondholders to actively plant saplings named after them. The local farmers purchasing those VCSBs will be the carers of those treasured saplings. The surplus capital investment on the environment is not new in the ancient civilization of Sri Lanka [60]. Many have promoted it as sustainable solutions [61, 62]. As pointed out by [62], it is investing in nature-based solutions.

## 4. Conclusion

This study revealed that open dumping and solid waste transportation are significant sources of GHG emissions in the LAs of Vavuniya. The Pampaimadu dumpsite, which is the largest, is affecting the populations living near the dump. Only those communities are aware of the present management system, but most of the populations are unaware of the constraints faced by the LAs. Inevitably, the impacts of solid waste on the environment and the implications of climate change and global warming are not explicitly known. According to the IPCC recommendations, the integration of solid waste composting reduces nearly 57 percent of GHG emissions when compared to the BAU approach. The introduction of electric vehicles cut the emissions from the solid waste transportation sector. The initial investment is high. However, the fuel cost cut off when using electric vehicles. LAs are better placed to fulfil the NDC. Because the project is technically and financially feasible, catalyzing the process of achieving carbon net zero goals. Moreover, the investments for implementing CSRP can be directly derived from saving on fossil fuel use, which is also avoidance. Such climate actions are contrary to the belief that developing countries will take longer time to peak emissions and that emission reductions are undertaken based on equity and in the context of sustainable development while eradicating poverty, which are critical development priorities for many developing countries. The RS is beneficial in achieving the national solid waste management policy statements aimed at reducing the amount of organic waste sent to landfills. NDCs often emphasize the importance of international cooperation and collaboration in achieving emission reduction goals. The LAs can get support from international partners, organizations, and funding mechanisms to implement emissions reduction measures in the waste management sector. The responsibilities of the LAs will be greater; thus, they need adequate support from the Provincial Council and the Central Government to implement the emission reduction strategic plan.

## Figures and Tables

**Table 1:** Baseline information of the LAs of Vavuniya [16].

Name of the local authority	Number of ward places	Name of the ward places	Estimated population in 2023	Population density per sq. km
Vavuniya Urban Council	10	Thandikulam, Pattanichchipuliyankulam, Pandarikulam, Vairavapuliyankulam, Kudiyiruppu, Kadai Veethy, Moonrumurippu, Rambaikulam, Sinnaputhukulam, Kovilkulam	51,933	2415

Vavuniya South Tamil Pradesiya Shaba	13	Marandamadu, Semamadhu, Echchannkulam, Sasthirikoolankulam, Poovarasankulam, Salambaikkulam, Pampaimadu, Marekkarampalai	85,763	150.77
		Maharambaikulam, Nelukulam, Kandapuram, Samalankulam, Asikulam		

Vengallacheddikulam Pradesiya Shaba	10	Periyathampanai- Piramanalannkulam, Andiyapuliyankulam, Kurukkalputhukkulam- Kanthasaminagar, Pavatkulam, Sooduventhapulavu	30,503	77.26
		Periyanpuliyankulam, Mugaththankulam- cheddikulam, Muthaliyankulam- Kristhavakulam, Neriyakulam, Sinnasippikulam		

Vavuniya North Pradesiya Shaba	14	Kanagarayankulam North – Mannakulam, Kanagarayankulam South, Sinnaadampan – Nainamaduwa, Kulavisuddan – Marailuppai, Nedunkerny, Olumadu – Mamadu, Puliyankulam North and South, Paranthan – Anandar, Pulliyakulam, Unchalkatty – Maruthoddai, Paddikudiyiruppu – Katkulam, Gajabapura Monarawewa, Kalayanapura Gammanaya, Ethawetunawewa, Sampath Nuwara	16,230	21.75

Vavuniya South Sinhala Pradesiya Shaba	9	Mamaduwa, Nedunkulam, Mahakachchakodiya, Madukanda, Awaranthulawa, Periya Ukkulama, Erattaperiyakulama, Alagalla, Maradammaduwa	24,600	104.28

**Table 2:** Solid waste management approaches as “BAU” and “RS.”

Business as usual (BAU)	Recommended strategies (RS)	Reason/s for developing the recommended strategies
Open dumping of degradable waste	Composting	The anaerobic decomposing of degradable waste from open dumping is a critical source of GHG emissions. Composting also produces GHG emissions. However, when compared to open dumping, it is less. Composting is an economically feasible option, and it is in demand in this region [18]

Gasoline solid waste collection vehicles	Solar panels will be used to charge electric vehicles for solid waste collection and transportation	The initial investment cost is high. However, it is recommended to charge the electric vehicles by the solar panel, which will cut off future fuel expenses
		Vavuniya is located in the dry zone of Sri Lanka, where the solar radiation is enough throughout the year to charge the vehicles

Emissions from dumpsites and vehicles before 2025 and the emissions from dumpsites and composting after 2025	Carbon sink reforestation program	Even to stop the open dumping of degradable waste in 2025, GHG emissions will be produced until they reach saturation. Reforestation is recommended to sequestrate already emitted GHG emissions dumpsites and vehicles. In addition, emissions from composting and open dumping will be after 2025

**Table 3:** GHG emissions from dumpsites.

Name of the dumpsite	GHG emission in 2023 (tons of CO₂ equivalents in 2023)	Cumulative GHG emission in 2023 (tons of CO₂ equivalents up to 2023)	Expected GHG emission in 2040 (tons of CO₂ equivalents in 2040)	Cumulative GHG emission in 2040 (tons of CO₂ equivalents up to 2040)
Pampaimaddu	1821.73	27118.48	2453.66	63908.08
Periyakattu	219.5	1965.8	338.58	6849.74
Sooduventhan	19.13	88.99	40.53	630.42
Puttkulam	9.57	44.5	20.26	315.21

**Table 4:** Input parameters to calculate the GHG emission from SWDS.

Factor	Symbol	Value	
Estimated population	P_2023_ and P_2040_	P_2023_	P_2040_
		VUC–51,933	VUC–64,775
		VSTPS–85,763	VSTPS–1,06,972
		VCPS–30,503	VCPS–38,046
		VNPS–16,230	VNPS–20,243

Solid waste generation (tons/day)	SWG_2023_	SWG_2023_	SWG_2040_
	SWG_2040_	VUC–31.16	VUC–38.87
		VSTPS–33.42	VSTPS–42.79
		VCPS–12.20	VCPS–15.22
		VNPS–6.49	VNPS–8.09

Solid waste collection (tons/day)	SWC_2023_	SWC_2023_	SWC_2040_
	SWC_2040_	VUC–9.23	VUC–11.52
		VSTPS–3.0	VSTPS–3.84
		VCPS–2.05	VCPS–2.56
		VNPS–0.30	VNPS–0.38

**Table 5:** GHG emission from solid waste collection vehicles.

Factor	Symbol	Value	
Fuel consumption (L/Day)	FC_2023_	FC_2023_	FC_2040_
	FC_2040_	VUC–46.19	VUC–57.61
		VSTPS–20.53	VSTPS–25.6
		VNPS–8.21	VNPS–10.24
		VCPS–12.32	VCPS–15.36

GHG emissions from vehicles (tones of CO₂ equivalents per year)	GHG_V_	GHG_V 2023_	GHG_V 2040_
		VUC–41.348	VUC–51.57
		VSTPS–18.377	VSTPS–22.921
		VNPS–7.351	VNPS–9.1686
		VCPS–11.026	VCPS–13.752

Energy consumption of the vehicles (kW/day)	ECv	ECv_2023_	ECv_2040_
		VUC–2935.55	VUC–3661.45
		VSTPS–1304.68	VSTPS–1627.33
		VNPS–521.89	VNPS–650.93
		VCPS–782.81	VCPS–976.39

**Table 6:** GHG emission reduction at dumpsites compared to BAU.

Name of the dumpsite	GHG emission in 2040 (tons of CO_2_–eq./year)	GHG emission reduction in 2040 (%)
Pampaimaddu	187.98	89.17
Periyakattu	23.22	88.38
Sooduventhan	2.17	85.57
Puttkulam	1.09	85.50

**Table 7:** Required number of electric tractors and solar panels.

Parameters	VUC	VSTPS	VNPS	VCPS
Number of electric tractors (NV)	NV_2023_-4	NV_2023_-2	NV_2023_-1	NV_2023_-1
	NV_2040_-5	NV_2040_-2	NV_2040_-1	NV_2040_-2

Required solar panel (R_SP_)	R_SP2023_-2	R_SP2023_-1	R_SP2023_−1	R_SP2023_-1
	R_SP2040_-3	R_SP2040_-2	R_SP2040_−1	R_SP2040_-1

**Table 8:** Comparison of GHG emission and required area for tree plantation.

Name of the LA	Comparison of cumulative GHG emission in tons in 2040							Required area (hectares)
	Dumpsites		Composting	Solid waste collection vehicles		Total emission at LA		
	BAU	RS	RS	BAU	RS	BAU	RS	
VUC	48777.87	34188.59	6608.06	1649.8	899.93	50427.7	41696.58	109.73
VSTPS	15130.2	10604.82	2049.73	733.24	399.97	15863.4	13054.51	34.35
VCPS	6849.74	4148.01	1223.72	362.38	162.42	7212.13	5534.15	14.56
VNPS	945.63	439.06	229.45	196.97	63.66	1142.6	732.17	1.93
District	71703.45	49380.47	10110.96	2942.4	1525.97	74645.8	61017.41	160.57

**Table 9:** Capital costs for establishing solar electrification of waste collections and carbon sink bonds.

Capital costs				
Description	Qty	Units	Rate (USD)	Amount USD
Electric tractors	8	40 kW	8,940	71,523
Solar panels	6	100 kW	57,947	347,682
Plant propagation laboratory	1		33,113	33,113
Nurseries	3		39,735	119,205
Subtotal				571,523
Contingencies (2%)				11,430
Project development and supervision (3%)				17,146
Sub-total				600,099
Working capital in the year 0				6,623
Total capital costs				606,722
Vavuniya carbon sink bonds				183,208
Total investment				789,929

**Table 10:** Schedule of loan capital and interest in USD.

Long term loan		Year 0	Year 1	Year 2	Year 3	Year 4	Year 5

Loan (1) opening balance		606,722	507,342	398,025	277,776	145,502	0
Capital repayments on loan (1)			99,380	109,317	120,249	132,274	145,502
Total capital repayments			99,380	109,317	120,249	132,274	145,502
Draw downs							
Loan closing balance		606,722	507,342	398,025	277,776	145,502	0
Loan (1) interest	10%		60,672	50,734	39,802	27,778	14,550
Total interest			60,672	50,734	39,802	27,778	14,550
Total loan repayment			160,052	160,052	160,052	160,052	160,052

**Table 11:** Summary of expenditures in USD.

Depreciation	Year 1	Year 2	Year 3	Year 4	Year 5	Year 6	Year 7	Year 8	Year 9	Year 10
Cost of operations	12,725	13,361	14,029	14,730	15,467	16,240	17,052	17,905	18,800	19,740
Depreciation	11,911	11,911	11,911	11,911	11,911	11,911	11,911	11,911	11,911	11,911
General insurance	383	345	310	279	251	226	204	183	165	148
Total expenditure	25,018	25,616	26,250	26,920	27,629	28,377	29,167	29,999	30,876	31,799

**Table 12:** Revenue in USD.

Year 0 VCSB	183,208									

Excess generations	Year 1	Year 2	Year 3	Year 4	Year 5	Year 6	Year 7	Year 8	Year 9	Year 10

VUC	0.991	0.965	0.939	0.912	0.884	0.857	0.829	0.800	0.771	0.742
VSTPS	0.11	0.10	0.08	0.07	0.06	0.05	0.03	0.02	0.01	0.00
VCPS	0.46	0.46	0.45	0.44	0.44	0.43	0.42	0.41	0.41	0.40
VNPS	0.64	0.64	0.63	0.63	0.62	0.62	0.61	0.61	0.60	0.60
Total for LAs	220.60	215.64	210.61	205.52	200.36	195.12	189.83	184.46	179.03	173.86
Hours per day	6	6	6	6	6	6	6	6	6	6
Annual generations	483,119	484,055	484,590	484,692	472,517	460,178	447,690	435,030	422,213	410,041
Tariff	0.12	0.12	0.12	0.12	0.12	0.12	0.12	0.12	0.12	0.12
Total from excess generations	57,974	58,087	58,151	58,163	56,702	55,221	53,723	52,204	50,666	49,205
Govt. Contribution @ 6.5% of fuel	2,282	2,431	2,589	2,757	2,936	3,127	3,330	3,547	3,778	4,023
Generations from fuel savings	8,958	23,888	35,832	47,776	59,720	71,664	83,608	95,552	107,496	119,440
Generations from VCSBs	62,316	83,088	83,088	83,088	83,088	83,088	83,088	83,088	83,088	83,088
Total revenue from generation	131,531	167,493	179,660	191,784	202,446	213,101	223,749	234,391	245,027	255,756

**Table 13:** Working capital in USD.

Year		Year 0	Year 1	Year 2	Year 3	Year 4	Year 5	Year 6	Year 7	Year 8	Year 9	Year 10

Incremental working capital	40%	6,623	5,261	6,700	7,186	7,671	8,098	8,524	8,950	9,376	9,801	10,230

**Table 14:** Projected profit and loss accounts in USD.

Profit and loss	Year 1	Year 2	Year 3	Year 4	Year 5	Year 6	Year 7	Year 8	Year 9	Year 10
Revenue	131,531	167,493	179,660	191,784	202,446	213,101	223,749	234,391	245,027	255,756
Cost of sales										
Cost of operations	12,725	13,361	14,029	14,730	15,467	16,240	17,052	17,905	18,800	19,740
Depreciation	11,911	11,911	11,911	11,911	11,911	11,911	11,911	11,911	11,911	11,911
General insurance	383	345	310	279	251	226	204	183	165	148
Total cost of sales	25,018	25,616	26,250	26,920	27,629	28,377	29,167	29,999	30,876	31,799
Gross profit	106,512	141,877	153,410	164,864	174,818	184,723	194,583	204,392	214,152	223,957
Less: Overheads										
Amortization of preoperational expenditures	4,967									
Total overheads cost	4,967	-	-	-	-	-	-	-	-	-
Operating profit	101,545	141,877	153,410	164,864	174,818	184,723	194,583	204,392	214,152	223,957
Less: loan interest	60,672	50,734	39,802	27,778	14,550					
Profit before tax	40,873	91,143	113,607	137,086	160,267	184,723	194,583	204,392	214,152	223,957
Less: corporation tax		—	—							
Profit after tax	40,873	91,143	113,607	137,086	160,267	184,723	194,583	204,392	214,152	223,957
Less: dividends	—	—	—	—	—	—	—	—	—	—
Retained profit/(loss)	40,873	91,143	113,607	137,086	160,267	184,723	194,583	204,392	214,152	223,957
Net profit B/f		40,873	132,016	245,623	382,710	542,977	727,700	922,283	1,126,675	1,340,827
Net profit C/f	40,873	132,016	245,623	382,710	542,977	727,700	922,283	1,126,675	1,340,827	1,564,784
Total operating profit	101,545	243,422	396,832	561,696	736,514	921,237	1,115,820	1,320,212	1,534,363	1,758,320
ROI	−83.26%	−59.88%	−34.59%	−7.42%	21.39%	51.84%	83.91%	117.60%	152.89%	189.81%

**Table 15:** Forecast of project cash flow in USD.

Project cash flow		Year 1	Year 2	Year 3	Year 4	Year 5	Year 6	Year 7	Year 8	Year 9	Year 10

Net income		40,873	91,143	113,607	137,086	160,267	184,723	194,583	204,392	214,152	223,957
Add: depreciation		11,911	11,911	11,911	11,911	11,911	11,911	11,911	11,911	11,911	11,911
Add: interest paid		60,672	50,734	39,802	27,778	14,550					
Operating cash profit		113,456	153,788	165,320	176,774	186,728	196,634	206,493	216,303	226,062	235,868
Less: increase in working capital		6,623	5,261	6,700	7,186	7,671	8,098	8,524	8,950	9,376	9,801
Interest and loan repayment		160,052	160,052	160,052	160,052	160,052					
Operating cash flow		(53,218)	(11,525)	(1,431)	9,536	19,005	188,536	197,969	207,353	216,687	226,066
Investment activities:	(606,722)	160,052	160,052	160,052	160,052	160,052					
Project cash flow	(606,722)	106,833	148,526	158,621	169,588	179,057	188,536	197,969	207,353	216,687	226,066
Net cash flow B/F		—	106,833	255,360	413,980	583,569	762,625	951,162	1,149,131	1,356,484	1,573,170
Net cash flow C/F		106,833	255,360	413,980	583,569	762,625	951,162	1,149,131	1,356,484	1,573,170	1,799,237
Internal rate of return	23.18%										
Payback period in years	4										
Operation cash flows	(606,722)	106,833	148,526	158,621	169,588	179,057	188,536	197,969	207,353	216,687	226,066
Cumulative cash flows	(606,722)	(499,888)	(351,362)	(192,741)	(23,153)	155,903	344,440	542,409	749,762	966,448	1,192,515

## Data Availability

The data that support the findings of this study have not been deposited in any public repository. However, they are available from the corresponding author upon reasonable request.
